# Pregnancy as a transition: First‐time expectant couples′ experience with alcohol consumption

**DOI:** 10.1111/dar.12973

**Published:** 2019-08-23

**Authors:** Solène Gouilhers, Yvonne Meyer, Sophie Inglin, Stéphanie Pfister Boulenaz, Céline Schnegg, Raphaël Hammer

**Affiliations:** ^1^ School of Health Sciences (HESAV) HES‐SO University of Applied Sciences and Arts Western Switzerland Lausanne Switzerland; ^2^ Independent Project Manager Geneva Switzerland

**Keywords:** alcohol, pregnancy, qualitative study, couples, transition

## Abstract

**Introduction and Aims:**

Most official healthcare guidelines apply the precautionary principle by recommending that pregnant women abstain from any alcohol consumption. However, a number of women continue drinking alcohol while pregnant. The aim of this study was to investigate couples′ experiences of the issue of alcohol consumption during pregnancy as a transitional process.

**Design and Methods:**

Thirty semi‐directive joint interviews were conducted with couples expecting their first child in Switzerland. Interviews were analysed thematically with the help of ATLAS.ti.

**Results:**

Couples endorsed the imperative of changing drinking habits and all the women reduced their alcohol consumption, although some reported difficulties. First, we identified three themes describing how couples experienced the woman′s change of drinking habits as a smooth transition: Internalisation of risk discourses, abstinence as a social norm and embodiment of alcohol aversion. Second, we emphasised four kinds of difficulties that couples encountered in their everyday lives: burden of risk discourses, conflicting advice, social occasions and desire for alcohol.

**Discussion and Conclusions:**

This paper makes a significant contribution by examining prenatal drinking change as a transition. In this conceptualisation, the change of alcohol consumption is a relational process that is shaped by multiple changes and social norms. Our findings have important implications for practice. First, health professionals should be aware of the difficulties women experience when they abstain from alcohol during pregnancy. Second, our findings suggest the importance of a patient‐centred approach that considers the role of the partner in supporting a pregnant woman′s change of alcohol consumption.

## Introduction

Maternal antenatal alcohol consumption has become a growing public health concern since the identification of foetal alcohol syndrome in the early 1970s 1. Heavy prenatal drinking detrimentally affects the unborn child and can cause brain damage and cognitive and behavioural impairment 2. Prenatal binge drinking has also been suggested to have adverse effects on child cognition 3. However, effects to the foetus related to the timing of maternal alcohol consumption, and to low and moderate drinking during pregnancy are contested in the literature 4. According to current evidence, there is no known safe amount of alcohol consumption during pregnancy 3. Like most Western countries, official healthcare guidelines in Switzerland apply the precautionary principle of recommending that women abstain from any alcohol consumption while pregnant 5. Although a majority of women abstain from alcohol once they become pregnant, a number of them continue drinking alcohol, albeit it at a substantially reduced rate 6. The estimated prevalence of alcohol consumption during pregnancy is higher in Switzerland than in several other European countries 7. According to a recent study, 10% of pregnant women in Switzerland reported drinking wine within the previous week 8. In the French‐speaking Switzerland, 35% to 40% of pregnant women reported drinking to some degree 9.

Over the past few years, a growing body of international research has explored why women use alcohol during pregnancy despite the abstinence advice. Quantitative studies have identified several predictors associated with maternal drinking, such as heavy alcohol use before pregnancy, domestic violence and higher income levels 10. Qualitative studies have shed light on the reasons and social contexts associated with occasional drinking and abstinence. They emphasise that a woman′s abstinence during pregnancy is based on a complex interplay of factors, such as expert information, fear of adverse outcomes, cultural norms and the advice of friends and parents 11, 12, 13, 14. A perceived openness to having an occasional drink during pregnancy is related to a woman′s lay understanding and experience of risk, cravings, social situations and drinking culture, as well as psychological and material stressors 11, 15, 16. However, few studies have addressed the issue of maternal drinking through the lens of pregnancy as a transition 17. As a complex process occurring at physical, emotional, relational and social levels 17, 18, transition to motherhood has been characterised by stress and vulnerability, as well as shift in roles and values. This paper focuses on pregnancy as a major adjustment period of lifestyle and health behaviours. This transition is strongly shaped by the social norms of motherhood, discourses of risk and public health recommendations, whereby pregnant women are encouraged to avoid any harmful situations or actions, such as drinking alcohol, for the sake of their foetuses 19, 20, 21. However, changing drinking habits is not necessarily a straightforward process for some women 22. Even though pregnant women are willing to change their drinking habits, they may face challenges in everyday life, including uncertainty about appropriate behaviour, due in part to both social influence and conflicting information 12, 23. Conceptualising pregnancy as a social transition also means considering the role played by other significant people in the woman′s life 17, 24. Several studies have pointed to the influence of family and friends upon pregnant women, conveying either abstinence advice or pressure to drink alcohol 14. Whereas some surveys emphasise the importance of the male partner on maternal use of alcohol 25, no qualitative study has specifically addressed the change in women′s drinking patterns from the couple′s perspective.

The aim of this study was to explore pregnant women′s and their partner′s experiences of the transition that occurs when they change their alcohol use by drawing upon qualitative interviews. In particular, this study examined how couples perceived their changing behaviour of alcohol consumption in their everyday lives and whether they experienced this change as a smooth transition or as a difficult one.

## Design and Methods

The study was based on semi‐directive joint interviews with a purposive sample of 30 couples expecting their first baby in French‐speaking Switzerland. The criteria for exclusion were: couples where the woman never drank alcohol before pregnancy, couples where the woman had been diagnosed with problematic consumption of alcohol and pregnancies with medical complications. Recruitment occurred in 2013–2014 through snowball sampling techniques and obstetrician and midwife networks. We chose to use joint interviews to explore how couples perceived and managed the issue of alcohol use during pregnancy. The advantage of joint interviews over separate interviews is to access the interactions between the two participants since they prompt one another 26. Moreover, joint interviews offer opportunities to illuminate shared experiences and meanings, decisions and disagreements about everyday health practices 26. Interviews examined both partners′ alcohol consumption habits before pregnancy and during pregnancy, as well as perceptions of and responses to alcohol consumption as a health‐related risk, including male partners′ involvement in the pregnancy.

Interviews took place when the women were between 21 and 39 weeks pregnant, and couples were interviewed at a place convenient to them by three trained researchers (Sophie Inglin, Stéphanie Pfister Boulenaz, Céline Schnegg). Interviews lasted an average of 63 min. Nineteen couples were married, and 11 were living together. Eighteen couples were Swiss, three were from other European countries, and nine were mixed couples. Despite our efforts to select couples from diverse social backgrounds, most participants in our sample had completed tertiary (21 women and 16 men) or secondary education (nine women and 12 men), thus limiting the generalisability of the findings. The mean age was 31 years for women and 34 years for men. With one exception, all couples described their pregnancy as planned. Women′s reported changes in drinking habits after pregnancy recognition included strict abstinence, abstinence with a few exceptions and significant reduction, meaning they consumed alcohol in small quantities with varying degrees of regularity.

All interviews were audio recorded and transcribed verbatim. We performed a thematic analysis with the help of ATLAS.ti, a qualitative data analysis software 27. Trustworthiness was enhanced by collaborative data analysis among the researchers in order to reach interpretative convergence 27. After thoroughly reading the transcripts to gain familiarity with the data, team members examined several interviews and independently produced an initial code list based on the research questions and themes emerging from the data. We held regular meetings to revise and finalise the codebook by combining codes and creating new ones and by addressing difficulties and disagreements in coding. The study was approved by the Research Ethics Committees of both cantons concerned. The couples received an information sheet describing the study, and prior to the interview, they signed an informed consent form that ensured anonymity and confidentiality. All participants′ names are pseudonyms. Quotes used in this article were translated from French to English.

## Results

All the couples interviewed endorsed the principle that drinking alcohol during pregnancy was harmful for the foetus. The women had accordingly altered their drinking habits while either attempting to get pregnant or after learning they were pregnant. Whereas the women and their partners referred to an obvious change, they also reported various concerns and practical difficulties when describing the process of adjustment in detail.

The results are organised in two parts that account for the complexity of alcohol use change during pregnancy. First, we highlight three key themes describing how couples experienced the woman′s change of drinking habits as a smooth transition. Second, we examine four key themes reflecting the difficulties the women faced when changing their drinking habits, including the ways the couples dealt with these difficulties.

### *Abstinence or significant reduction as an obvious change*

#### *Internalisation of risk discourses*

The first theme related to the abstinence or significant reduction as an obvious change was the internalisation of risk discourses characterised by the anticipation of adverse outcomes and avoidance of uncertainty 28. All the women and their partners perceived the reduction of alcohol consumption or the abstinence as necessary for ensuring the good health of the foetus. Therefore, most women considered the change they made to be effortless. Most of the women adopted a precautionary approach: ‘Thinking that in continuing to drink or to smoke you can inflict a birth defect [on your baby], it′s just terrible. How can you live with that?’ (Adeline). Other women made a few exceptions, only because they were sure that it would not harm the foetus: ‘We protect this baby so it′s true that all the substances, like medicine, alcohol, cigarette [are eliminated]. I′m doing my best to protect him from that’ (Judith). Judith claimed that as long as you are reasonable, you can have a ‘simple pleasure’, like enjoying a ‘drop of wine’. For Judith and other women, internalisation of risk discourses did not preclude the occasional consumption of a limited quantity of alcohol.

#### *Abstinence as a social norm*

The second theme was the change in alcohol consumption as a strong social norm. The majority of women and their partners considered abstinence a matter of common sense rather than an expert recommendation: ‘You know that if you get pregnant, you can′t drink anymore. It′s a little bit … popular I′d say’ (Flore). Violette explained that she did not need specific information to lower her consumption, but did so ‘by intuition’. Likewise, Sandrine linked her ease at becoming abstinent to the social norm: ‘It′s instinctive, I think that the fear of alcohol comes from the dominant discourse. If a woman who drinks while pregnant was not seen as the absolute taboo, I don′t think that it would come to me so naturally’.

Several women perceived abstinence as a shared norm among their relatives, which made it easier for them to change their alcohol consumption since they did not feel the need to justify themselves. Most of the couples had not discussed changing their alcohol consumption during pregnancy: ‘We didn′t discuss it.… I had the feeling that it was a normal behaviour’ (Marc). These couples were likely to view the transition as smooth. Furthermore, some women received active support from their partners or their relatives, for example, by being offered appealing alcohol‐free drinks.

#### *The embodiment of alcohol aversion*

The third theme related to the body′s reactions to alcohol as an unexpected facilitator of alcohol reduction. While several women viewed alcohol as a pleasure associated to their ordinary sociability, some of them experienced pregnancy symptoms, such as nausea and tiredness, that helped them to quit drinking. For example, Sandrine, who ‘liked to drink very much’, recounted: ‘The body is well made. The first four months, I didn′t feel like drinking and smoking because I had nausea and quickly got used to it’. Likewise, Cécile and Mathieu, who described themselves as ‘big drinkers’, associated alcohol with going out and good food. While Cécile sometimes missed alcohol in early pregnancy, she stated that her body started to react to alcohol and drinking was no longer a temptation: ‘Yesterday there was a barbecue in our village, and I had no desire to drink.… I can′t stand alcohol anymore’.

### *Difficulties in daily life*

#### *The burden of risk discourses*

The first theme, concerning the difficulties women encountered when attempting to change their drinking habits, pertained to anxiety generated by the burden of risk discourses. Several women experienced feelings of guilt and concern after consuming alcohol: ‘The exceptions I made, I remember them very clearly and I felt guilty.… I shouldn′t have’ (Elsa). Other couples experienced feelings of stress and uncertainty due to heavy drinking before pregnancy recognition. For example, Cécile recounted: ‘At New Year′s day, we were in a cottage … the poor [baby], I got heavily drunk!’. She and her husband were afraid of the possible consequences and sought reassurance from a family member.

In order to escape such feelings of stress‐related risky behaviours during the course of pregnancy, some women turned to strict abstinence. Limiting alcohol use and claiming to drink ‘responsibly’ was another means of dealing with feelings of guilt. While professional advice could be reassuring, relatives or friends also could alleviate women′s fears by downplaying the risk. For example, Stéphanie ate fondue containing alcohol while pregnant, which made her feel anxious: ‘At one point I got into a panic, then my friends told me, “Calm down. It′s cooked, so the alcohol evaporates”.… I needed to be reassured. That eased my mind’. Male partners also played significant roles in trying to alleviate women′s concerns. José, for example, convinced his wife that small exceptions to abstaining from alcohol use were ‘not the end of the world’. In rare cases, couples reported tensions regarding the use of alcohol during pregnancy. For example, Bruno, who stated that he had ‘a very low tolerance [for risk]’, irritated Sandrine who felt she was being watched when drinking a small quantity of alcohol: ‘It really gets on my nerves … it′s as if I were a bit irresponsible, I don′t find that respectful’.

#### *Conflicting advice*

The second theme related to the difficulties created by conflicting or confusing advice about alcohol consumption during pregnancy. Several couples reported that they were unclear about health provider′s recommendations regarding a safe amount of alcohol intake during pregnancy. Some of them experienced contradictory information as a cause for concern about the appropriate behaviour: ‘Physicians say, “really nothing”, but some of them say, “a champagne flute on Christmas”, but they didn′t clearly say a certain amount that we can rely on’ (Bruno). Like other couples, Violette and Marc talked about the ‘fuzzy’ information on the web where one can read ‘everything and its opposite’.

As a frequent response to such uncertainty, interviewees claimed that each couple has to define its own way of managing the risk, resulting in two distinct strategies. The first one was to err on the side of caution, bringing some couples to ‘choose zero [alcohol]’ (Lilian). The second strategy consisted of finding a compromise between the contradictory advice they encountered: ‘The problem is that we hear a bit of everything.… We learn a little bit of information everywhere, and we say, “All right, let′s split the difference. We diminish, or we drink a sip, and that′s all”’ (Cécile). Like Cécile, many women and their partners reported that additional stress was produced by the need to identify behaviour that is safe or harmful for the baby.

#### *Social occasions*

Consuming alcohol as a part of social life was the third theme reflecting the difficulties couples encountered. The first aspect of these difficulties concerned the lack of alcohol in connection with drinking habits. Several women experienced abstinence from alcohol in social events as a strong break from their prior practices. Some of them recounted how much their non‐use of alcohol made them feel excluded in certain social occasions: ‘For me, wine and dinner go hand in hand. It′s a pleasure.… After a while you′re disconnected from the party.… It′s excluding a little bit.… I miss drinking a lot!’ (Judith). In order to overcome such frustrations, women adopted various strategies, often with their partner′s help. Some chose to drink non‐alcoholic beer as if they were sharing a glass of alcohol with people. Others viewed abstinence as a temporary sacrifice by perceiving pregnancy as a short period of their lives. Still others found support from their peers: ‘Fortunately, I have other friends who are pregnant’ (Sylvie).

The second aspect referred to peer pressure to drink in social occasions, such as at parties or birthdays. The social norm of drinking was especially difficult to manage when pregnancy was not revealed yet to others: ‘It′s tough when [the pregnancy] is secret! There is really a social pressure regarding alcohol. It′s crazy!’ (Céline). Couples developed tricks, such as discreetly exchanging glasses. Some male partners actively supported their wives to resist peer pressure: ‘When we went out with friends to eat, some of them often said, “But Manuella, you can taste, drink a little bit”. It tended to upset her. I took this position: “We must respect her choice. She has decided to not drink!”’ (Vincent). Finally, reducing one′s social life, especially aperitifs or nights out, was another strategy for avoiding peer pressure to drink.

#### *Desire for alcohol*

The fourth theme reflecting the difficulties related to the women′s desire for alcohol. Unlike the previous theme, the challenge was not connected with the social meaning of alcohol, but rather with the product itself. In our study, several women strongly associated alcohol, especially a good wine or a cold beer, with pleasure. For example, Manuella has been abstinent since the beginning of her pregnancy and said: ‘I miss drinking a little glass of wine, it′s a treat that I had’. Efforts to reduce consumption of or abstain from alcohol could sometimes be disrupted by occasional cravings in specific situations, such as meals and parties. Léonie′s angry reaction to a friend′s attitude challenging her abstinence choice revealed her strong temptation to taste alcohol: ‘She pisses me off to put that in my head! It′s generating a desire for alcohol! One starts thinking, “Why not?”, “I could taste” or “It′s not too bad”.… You end up tasting it’.

Some women chose to manage their desire for alcohol by drinking a predetermined amount in specific situations to minimise the risk while enjoying the pleasure. Others chose abstinence as the easiest option: ‘It would be all the more frustrating to drink a full glass of wine and to want another one! So, I thought that it would be better to put that aside.… It was a way to protect myself, to avoid frustrations!’ (Adeline). Several women also relied on their partner′s support to help them handle feelings of temptation. While most of the male interviewees did not stop drinking during the women′s pregnancies, some of them had slightly altered their drinking pattern to ‘keep [their partners] away from temptation’ (Alan). In a few cases, women were unsatisfied with their partner′s support, as illustrated by Céline who felt it was ‘a rotten thing’ that her partner once opened a wine bottle at home in her presence.

## Discussion

Based on qualitative interviews with 30 first‐time expectant couples, this study makes a significant contribution to the literature on maternal drinking by examining prenatal alcohol consumption as part of the transition to parenthood. Within the transition framework 17, the change in alcohol consumption falls within the broader context of multiple adaptations and transformations 18. Specifically, the norm of alcohol reduction or abstinence is embedded in a larger set of prescribed behaviours attached to the pregnant woman′s role to protect her baby: quitting smoking, avoiding certain types of food, engaging in medical monitoring, etc. 19, 21, 29. Furthermore, pregnancy as a transition suggests that the change in alcohol consumption is a relational process that includes the partner, rather than an individual process. Joint interviews allowed us to explore how couples dealt with alcohol consumption during this period of transition. Consistent with other findings 15, couples in our study were convinced that pregnant women needed to reduce or stop alcohol consumption. Indeed, all women had changed their drinking habits for the sake of their foetuses.

Most of the couples described the change in alcohol consumption as a normal adaptation rather than a rational and individual decision, in line with prior research 15. Our findings highlighted three key themes reflecting perceptions of abstinence or reduced alcohol consumption as an obvious change. First, risk discourses were highly internalised by most of the women and their partners. Most often, they opted for a ‘better safe than sorry’ approach 12, 30. Second, couples framed their avoidance of alcohol during pregnancy as a common‐sense decision that was likely to be shared among their relatives and in the couple relationship, which is consistent with previous studies 25. Consequently, the issue of alcohol consumption was not a matter of discussion 31. Third, physical aversion to alcohol due to symptoms of pregnancy helped several women become abstinent.

Contrary to a recent Australian study 15 in which women did not report difficulties, we identified four key themes reflecting the challenges that women faced in implementing drinking changes in their everyday lives. First, we found that feelings of guilt and anxiety are associated with the pervading discourses of risk 20. Such feelings have been linked to the contemporary cultural context characterised by the moral norms of motherhood and risk avoidance 15, 19, 21, 29. Couples faced a second challenge from conflicting advice about appropriate changes in drinking habits, which was a source of confusion, as prior research has shown 12, 14, 15, 30. These difficulties resonate with studies indicating that the discourses of risk, including public health messages and scientific knowledge, while being thought of as a resource for control over health, may generate unintended consequences, such as uncertainty, undue anxiety, increased sense of vulnerability and moralisation of pregnant women′s responsibility 19, 21, 28, 32. Third, despite strong social norms in favour of abstinence, our findings confirm that pregnant women may be subject to peer pressure to drink, especially when the pregnancy is not yet visible. This is consistent with studies that highlight the difficulty of being abstinent within the context of a drinking culture 13, 23, and show that pregnant women are confronted with contradictory social norms regarding their alcohol consumption 12. We have identified a fourth struggle, which is that some women missed the taste of alcohol and the pleasure associated with it, admitting that such feelings challenged their willingness to abstain.

Women described different consumption practices that avoided guilt or frustration. Similar to other studies, some women became strictly abstinent, while others defined their own limits of alcohol consumption 15, 23. Such responses to risk, labelled as ‘responsible drinking practices’ 33 by the women themselves, have been found in other studies 11, 12, 34. Consistent with prior research 15, our findings underline that the responsibility of changing drinking habits rested mainly on the shoulders of women. Yet, we have also shown that male partners can play a significant role in helping them to handle the struggles associated with reducing their alcohol intake. In this respect, only few couples expressed conflicts or disagreements resulting from difficulties in managing alcohol use in daily life.

Our findings have important implications for guiding health professionals to address the issue of alcohol consumption during pregnancy. Contrary to an Australian qualitative study 23, women in our study rarely mentioned health professionals as important resources for helping them manage anxiety and guilt. This is consistent with a Swiss study indicating that one‐third of women did not receive any information regarding alcohol consumption during pregnancy from health professionals 35. In our sample, most of the couples were not satisfied by the information provided by their midwife or gynaecologist, reporting that they never discussed practical ways to reduce alcohol consumption nor received support to deal with conflicting advice. Nevertheless, several couples would have liked to receive clearer messages regarding abstinence from their health professionals, consistent with official guidelines. We propose three recommendations to enhance the support that health professionals can provide to women and their partners.

First, health professionals should be aware that all women, including those who subscribe to the recommendation of abstinence and choose to abstain, can face difficulties that are not necessarily disclosed to them. As argued by prior research 36, 37, the practitioner′s ability to initiate an open and non‐judgmental discussion with women about their alcohol consumption habits is essential to provide adequate support, especially to those with substance use issues 22. Second, our findings encourage health professionals to view pregnancy as a complex transition. This period of instability is shaped by multiple changes and different norms that may give rise to ambivalent feelings toward abstinence. Pregnant women do not change their drinking habits overnight; it is a contextualised process happening all throughout the pregnancy with multiple adaptations in accordance with specific situations. A patient‐centred approach should be aware of this complexity and consider women′s lifestyles, existing resources, difficulties and the couples′ perspectives 23, 38. For example, health professionals could pay attention to both partners′ drinking habits before pregnancy, such as alcohol as a social identity marker or as a response to stressors 16. Health professionals can also pay attention to conflicting advice and social norms about antenatal alcohol consumption, as well as to women′s feelings of anxiety and guilt, even when they consume very small amounts of alcohol 11, 15, 30. Third, our findings suggest enhancing the involvement of the partners of pregnant women. Whereas the responsibility to change alcohol consumption habits rests on the women′s shoulders, most partners aimed to be supportive 36, and several women deemed their support helpful in daily life. In 2015, the WHO published recommendations regarding maternal and neonatal health 39 that highlighted the father′s involvement during pregnancy, birth and post‐partum as a central factor of health. Health professionals could therefore encourage partners′ supportive attitudes towards pregnant women′s change of alcohol use.

### *Strengths and limitations*

This study′s strength lies in the richness of the data collected from interviews conducted jointly with pregnant women and their partners, providing access to both partners′ perspectives and their interactions 26. As the couples in our sample had rarely discussed the topic of alcohol use during pregnancy, joint interviews helped explicate both partners′ views, tacit knowledge and normative values about this matter, which ‘are more likely to remain invisible in individual accounts’ 26 (p. 1644). While the influence of social desirability and power relations between partners cannot be excluded, most couples expressed their views openly, including the uncertainties and difficulties that they encountered when adjusting their lifestyles. Our findings cannot be generalised to all first‐time expectant couples′ experiences with alcohol use in Switzerland in two respects. First, couples from middle and high education levels were over‐represented in our sample. Further research should therefore focus on couples with lower socioeconomic status. A second limitation of our study is the exclusion of pregnant women with problematic alcohol consumption. However, some of our findings are arguably transferable to this particular group of women. Indeed, a study focussing on vulnerable women also reported maternal responsibility, physical aversion to alcohol and supportive personal relationships as facilitators of alcohol reduction 22. Certain difficulties, such as drinking culture and peer pressure to drink, have also a more general scope and are likely to be exacerbated and more burdensome for pregnant women with substance use issues and socioeconomic hardship 16, 22. Moreover, separate interviews could be used rather than joint interviews to research how interpersonal pressures are exercised in couples, especially for those in relationships characterised by conflict or violence.

## Conclusion

This qualitative study explored how couples managed alcohol use during pregnancy. Our findings show that abstinence or significant reduction of alcohol consumption during pregnancy was a shared norm between the two partners. We identified how couples experienced changes in drinking patterns as a smooth transition, and also highlighted the difficulties that women encountered in abstaining from or reducing alcohol consumption in their everyday lives. Health professionals should be aware of these common difficulties and support the women and their partners during pregnancy as a complex social transition.

## Conflict of Interest

The authors have no conflicts of interest.

## References

[1] Jones KL , Smith DW , Ulleland CN , Streissguth P . Pattern of malformation in offspring of chronic alcoholic mothers. Lancet 1973;1:1267–71. 4126070 10.1016/s0140-6736(73)91291-9

[2] Hoyme HE , Kalberg WO , Elliott AJ *et al* Updated clinical guidelines for diagnosing Fetal alcohol spectrum disorders. Pediatrics 2016;138:1–18. 10.1542/peds.2015-4256 PMC4960726 27464676

[3] Flak AL , Su S , Bertrand J , Denny CH , Kesmodel US , Cogswell ME . The association of mild, moderate, and binge prenatal alcohol exposure and child neuropsychological outcomes: a meta‐analysis. Alcohol Clin Exp Res 2014;38:214–26. 23905882 10.1111/acer.12214

[4] O′Leary CM , Bower C . Guidelines for pregnancy: what′s an acceptable risk, and how is the evidence (finally) shaping up? Drug Alcohol Rev 2012;31:170–83. 21955332 10.1111/j.1465-3362.2011.00331.x

[5] Federal Office of Public Health . Fiche d′information: alcool et grossesse. Berne: Office fédéral de la santé publique, 2011.

[6] Tran NT , Najman JM , Hayatbakhsh R . Predictors of maternal drinking trajectories before and after pregnancy: evidence from a longitudinal study. Aust N Z J Obstet Gynaecol 2015;55:123–30. 25537524 10.1111/ajo.12294

[7] Mårdby A‐C , Lupattelli A , Hensing G , Nordeng H . Consumption of alcohol during pregnancy‐a multinational European study. Women Birth 2017;30:207–13. 10.1016/j.wombi.2017.01.003 28111037

[8] Bornauser C , Lötscher KC , Seifert B , Simoes‐Wüst AP . Diet, medication use and drug intake during pregnancy: data from the consecutive Swiss health surveys of 2007 and 2012. Swiss Med Wkly 2017;147:w14572. 29282704 10.4414/smw.2017.14572

[9] Meyer‐Leu Y , Lemola S , Daeppen J‐B , Deriaz O , Gerber S . Association of moderate alcohol use and binge drinking during pregnancy with neonatal health. Alcohol Clin Exp Res 2011;35:1669–77. 21554334 10.1111/j.1530-0277.2011.01513.x

[10] Skagerstróm J , Chang G , Nilsen P . Predictors of drinking during pregnancy: a systematic review. J Women's Health 2011;20:901–13. 10.1089/jwh.2010.2216 PMC3159119 21671775

[11] Hammer R , Inglin S . ‘I don′t think it′s risky, but…’: pregnant women′s risk perceptions of maternal drinking and smoking. Health Risk Soc 2014;16:22–35.

[12] Loxton D , Chojenta C , Anderson AE , Powers JR , Shakeshaft A , Burns L . Acquisition and utilization of information about alcohol use in pregnancy among Australian pregnant women and service providers. J Midwifery Womens Health 2013;58:523–30. 26055614 10.1111/jmwh.12014

[13] Jones SC , Telenta J . What influences Australian women to not drink alcohol during pregnancy? Aust J Prim Health 2012;18:68–73. 22394665 10.1071/PY10077

[14] Elek E , Harris SL , Squire CM *et al* Women′s knowledge, views, and experiences regarding alcohol use and pregnancy: opportunities to improve health messages. Am J Health Educ 2013;44:177–90. 28261370 10.1080/19325037.2013.768906 PMC5332134

[15] Holland K , McCallum K , Walton A . ‘I′m not clear on what the risk is’: women′s reflexive negotiations of uncertainty about alcohol during pregnancy. Health Risk Soc 2016;18:38–58.

[16] Watt MH , Eaton LA , Choi KW *et al* “It′s better for me to drink, at least the stress is going away”: perspectives on alcohol use during pregnancy among south African women attending drinking establishments. Soc Sci Med 2014;1:119–25. 10.1016/j.socscimed.2014.06.048 PMC4117814 24997441

[17] Kralik D , Visentin K , Loon AV . Transition: a literature review. J Adv Nurs 2006;55:320–9. 16866826 10.1111/j.1365-2648.2006.03899.x

[18] Meleis AI , Sawyer LM , Im E , Messias DKH , Schumacher K . Experiencing transitions: an emerging middle‐range theory. Adv Nurs Sci 2000;23:12–28. 10.1097/00012272-200009000-00006 10970036

[19] Lupton D . Risk and the ontology of pregnant embodiment In: Lupton D, ed. Risk and sociocultural theory: new directions and perspectives. Cambridge: Cambridge University Press, 1999:59–85.

[20] Kangasniemi M , Blomberg K , Korhonen A . Mothers′ perceptions of their health choices, related duties and responsibilities: a qualitative interview study. Midwifery 2015;31:1039–44. 26194325 10.1016/j.midw.2015.06.013

[21] Hammer RP , Burton‐Jeangros C . Tensions around risks in pregnancy: a typology of women′s experiences of surveillance medicine. Soc Sci Med 2013;93:55–63. 23906121 10.1016/j.socscimed.2013.05.033

[22] Latuskie KA , Andrews NCZ , Motz M *et al* Reasons for substance use continuation and discontinuation during pregnancy: a qualitative study. Women Birth 2019;32:e57–64. 29673617 10.1016/j.wombi.2018.04.001

[23] Meurk CS , Broom A , Adams J , Hall W , Lucke J . Factors influencing women′s decisions to drink alcohol during pregnancy: findings of a qualitative study with implications for health communication. BMC Pregnancy Childbirth 2014;24:246. 10.1186/1471-2393-14-246 PMC4122767 25060554

[24] Högberg H , Skagerström J , Spak F , Nilsen P , Larsson M . Alcohol consumption among partners of pregnant women in Sweden: a cross sectional study. BMC Public Health 2016;16:694–703. 27484750 10.1186/s12889-016-3338-9 PMC4971635

[25] van der Wulp NY , Hoving C , de Vries H . Partner′s influences and other correlates of prenatal alcohol use. Matern Child Health J 2015;19:908–16. 25087003 10.1007/s10995-014-1592-y

[26] Polak L , Green J . Using joint interviews to add analytic value. Qual Health Res 2016;26:1638–48. 25850721 10.1177/1049732315580103

[27] Patton MQ . Qualitative research & evaluation methods, 3rd edn. London: Sage, 2002.

[28] Giddens A . The consequences of modernity. Cambridge: Polity Press, 1990.

[29] Lupton D . ‘Precious cargo’: foetal subjects, risk and reproductive citizenship. Crit Public Health 2012;22:329–40.

[30] France K , Donovan R , Henley N *et al* Promoting abstinence from alcohol during pregnancy: implications from formative research. Subst Use Misuse 2013;48:1509–21. 23819741 10.3109/10826084.2013.800118

[31] Baxter LA , Hirokawa R , Lowe JB , Nathan P , Pearce L . Dialogic voices in talk about drinking and pregnancy. J Appl Commun Res 2004;32:224–48.

[32] Lowe PK , Lee EJ . Advocating alcohol abstinence to pregnant women: some observations about British policy. Health Risk Soc 2010;12:301–11.

[33] Waitt G , Clement S . Women drinking alcohol: assembling a perspective from a Victorian country town, Australia. Gend Place Cult 2016;23:1121–34.

[34] Jayne M , Valentine G , Holloway SL . Emotional, embodied and affective geographies of alcohol, drinking and drunkenness. Trans Inst Br Geogr 2010;35:540–54.

[35] Dravta J , Gross K , Späth A , Zemp SE . SWIFS ‐ Swiss infant feeding study: a national study on infant feeding and health in the child′s first year: executive summary. Basel: Swiss Tropical and Public Health Institute, 2014.

[36] Crawford‐Williams F , Steen M , Esterman A , Fielder A , Mikocka‐Walus A . “My midwife said that having a glass of red wine was actually better for the baby”: a focus group study of women and their partner′s knowledge and experiences relating to alcohol consumption in pregnancy. BMC Pregnancy Childbirth 2015;15:79. 25881173 10.1186/s12884-015-0506-3 PMC4389416

[37] Stone R . Pregnant women and substance use: fear, stigma, and barriers to care. Health Justice 2015;3:2.

[38] Shields MDSG , Candib MDLM . Women‐centered care in pregnancy and childbirth. New York: Routledge, 2010.

[39] World Health Organization . Recommendations on health promotion interventions for maternal and newborn health. Geneva: World Health Organization, 2015. 26180864

